# Personalised medicine in the UK: challenges of implementation and impact on healthcare system

**DOI:** 10.1186/gm545

**Published:** 2014-04-25

**Authors:** Anna Pokorska-Bocci, Mark Kroese, Gurdeep S Sagoo, Alison Hall, Hilary Burton

**Affiliations:** 1 PHG Foundation, 2 Worts Causeway, Cambridge CB1 8RN, UK

## Personalised medicine initiatives in the UK

In light of the increasing momentum towards personalised medicine and healthcare, driven by genomic science and a spectrum of technological and information tools [1], we discuss the challenges and impact of personalised approaches within the UK National Health Service (NHS). Various initiatives in the UK have attempted a shift from population-centred to individual-centred approaches and there have been some successful attempts to make healthcare systems more participatory and prevention-oriented.

In the UK, personalised approaches have featured on policy agendas for some time. In 2009, the House of Lords report on Genomic Medicine noted that using genomics to enable stratified use of medicines holds ‘the greatest potential for the healthcare sector’ but also presents ‘one of the biggest translational challenges’ [2]. Stratified medicine initiatives, co-financed by industry and government, have encouraged the pharmaceutical industry to embrace personalised treatments and devices. In 2011, the Technology Strategy Board published a strategic vision for making the UK the world leader in development and adoption of stratified medicine. In 2012, the potential emergence of stratified medicine across the entire healthcare system fuelled by genomic information was a central theme of the governmental Human Genomics Strategy Group report [3].

## Challenges for implementing personalised medicine in the UK

Despite the high-level political endorsement, personalised medicine remains elusive and several challenges remain. These include establishing a robust evidence base, changing practice within existing health services, and facilitating an increasingly participatory approach. Some of these challenges were analysed in the PHG Foundation response to the Genomic Medicine report and its report on next-generation sequencing [4,5]. The Academy of Medical Sciences noted a further set of economic drivers for adopting personalised approaches [6].

### Establishing the evidence base

A personalised approach necessitates tests and interventions that target increasingly finely divided subgroups requiring a valid evidence base incorporating genomic data. Genomics may contribute to personalised medicine by allowing refined knowledge of a diagnostic group or a subgroup characterised by its response to a treatment . Demonstrating the clinical utility and cost-effectiveness of expensive complex technologies in genetic diseases, and that the replacement test is superior to existing interventions in efficacy, cost-effectiveness or discriminatory power, are necessary steps for implementation. Many molecular tests have failed to satisfy these requirements [7].

As subgroup size diminishes, the challenge of establishing a valid evidence base will be correspondingly greater. Yet the scale of overall knowledge generation and its adequate storage and sharing in order to create scientific and clinical evidence is a challenge in itself and requires new infrastructures and processes.

Development of the evidence base is a vital part of the translation process. Crucial to this are prospective approaches that aim to further understand the occurrence and progression of disease and identify subgroups of patients sharing similar responses to treatments, such as the Stratified Medicine Initiatives, and strategies developed by the UK Medical Research Council and Cancer Research UK .

In the UK, evidence-based commissioning support for the introduction of new technologies is provided through National Institute for Health and Care Excellence (NICE) guideline development. NICE uses metrics such as the quality-adjusted life-years (QALY), which combines the quality of life gained from an intervention with the additional length of life gained. However, current health economics practice for NHS interventions does not include non-health outcomes, such as reproductive choice and impact on family members, in the context of genomics within QALY calculations [5]. This is in part due to the lack of cost data or values applied to these outcomes. Furthermore, there are wide-ranging methodological issues, including measuring outcomes and effectiveness, that may require resolution before the lack of evidence base can be addressed [8].

### Refining the organisation and operation of health services

Developing integrated and increasingly sophisticated molecular pathology and diagnostics, which are the prerequisites of personalised medicine, will pose challenges for health services. Interpreting detailed and complex findings and integrating these with phenotypic and ongoing clinical data, including outcomes, will require the development of electronic health records. In the UK, NHS Scotland has established an eHealth strategy to be implemented over the next five years that aims to enhance integration and availability of information, improve communication and enable people to be more active participants in their healthcare [http://www.ehealth.scot.nhs.uk/]. Patient pathways must then be developed that are technically flexible and responsive to individual patients’ needs. All this will require resources and in this financial climate will only be achievable if new practices are as, or more, clinically effective and cost-effective than existing technologies.

New approaches have to be ethical and acceptable to professionals and patients alike. The consent process will become more dynamic and flexible and the ability of patients to make autonomous decisions increasingly important. This may require clinical guidelines to be revised and decision support systems to be developed.

### Facilitating the shift to participatory medicine

A key characteristic of personalised medicine is the paradigm shift to the participatory healthcare model [9]. Facilitating greater awareness of available choices and providing increased control over health management creates novel pressures on health services and health professionals, but also imposes new duties upon individuals. Patients and the public will need to commit to the systematic capture and sharing of clinical data to build and fine-tune the knowledge base. This commitment will extend both to routinely collected health data and to sharing anonymised data through big data initiatives. Many personalised healthcare initiatives in the UK acknowledge that patients and the public should and will be involved in designing these new, more participatory services. The Clinical Reference Groups of NHS England provide a good example where patients and carers become stakeholders, with engagement ranging from being kept informed to participating in consultation processes.

An increasing number of drugs and available treatments will be directly linked with the patient undergoing a diagnostic test, showing the likelihood of benefit from that particular treatment. Increased patient participation in the management of their health brings a requirement for health literacy, enhanced use of modern communication tools, and readiness for dialogue with medical providers.

## Impact of personalisation on public health and the UK healthcare system

Whilst acknowledging these challenges, proponents of a personalised agenda should be encouraged by various developments within the UK. Personalisation may seem to be at odds with the population-based prevention agenda but actually the different approaches show synergy. UK prevention programmes such as healthy eating or reduction of tobacco consumption assume that the population is homogeneous and interventions that reduce the average risk will have a major effect. The complementary approach involves identification of a high-risk group for special preventive interventions. This principle is embedded in the UK NHS Health Check programme, for example, which uses conventional risk factors such as age, sex, body mass index, smoking status, and a range of physiological measurements to identify people requiring intensive preventive management. Addition of genetic biomarkers enables this approach to further fine-tune risk estimation. Risk can be stratified at an early age and across the whole risk spectrum [10]. Individuals who are low risk are not potentially harmed by preventive interventions and resources are targeted more efficiently.

Introducing genomics into population-based prevention programmes will not be simple. It will be important to ensure that individuals are not unduly worried by their risk status or confused by differential prevention advice, that those at high risk are not stigmatised by employers or insurers, and that people trust the systems that will handle their genetic information. Integral to the introduction of many aspects of personalised prevention in the UK will be involvement of the National Screening Committee, the body that advises ministers and the NHS in all four countries about all aspects of screening and supports the implementation of screening programmes. In particular, the Committee will need to address the principle of two-stage screening, where the first step involves determination of individual risk, based on biomarkers, family history and physiological measurement, before progressing to application of the screening intervention. Such a step involves a paradigm shift from the concept of screening programmes applied on an equal basis to large populations.

The UK 100,000 Genomes Project [http://www.genomicsengland.co.uk/100k-genome-project/] is a major initiative that will promote personalised medicine in the UK by providing a mechanism for developing new diagnostics and treatments, and explicitly linking these to clinical care. An important aspect of this has been the setting up of a strategic group under Health Education England that will take the lead in preparing the NHS workforce for the introduction of genomics. By engaging with all key stakeholders, including the public, this project has the potential to catalyse a system change.

## Conclusions

A plethora of scientific, technological and communication tools open new possibilities to improve healthcare by fine-tuning health management to individuals’ needs and preferences. However, a lot of work has still to be done to ensure that populations and individuals fully benefit from this new era. Health professionals, policy makers and other stakeholders need to work together to develop health services and involve patients and the public in the personalised medicine endeavour.

Establishing the evidence base and demonstrating the clinical utility of new tests or treatments will be crucial. Systematic use of electronic health records, employment of various methods to encourage participation of patients and clinicians in decision-making, and collection and sharing of health data will be prerequisites. Finally, we also note the importance of personalisation in the public health prevention agenda.

## Abbreviations

NHS: National Health Service; NICE: National Institute for Health and Care Excellence; QALY: quality-adjusted life-years.

## Competing interests

The authors declare that they have no competing interests.
